# User Satisfaction With Pregnancy Management Apps in Mainland China: User-Generated Content Analysis and Text Mining Study

**DOI:** 10.2196/78828

**Published:** 2025-08-21

**Authors:** Xiaoyi Jiao, Lu Jiang, Min Zhao, Junhao Ma, Yanwei Li, Tian Shen, Yongcheng Liu, Yue Hu, Zhengyang Lu, Mengyao Xing, Jun Liang, Peng Xiang, Jianbo Lei

**Affiliations:** 1 School of Public Health, Zhejiang University Hangzhou, Zhejiang Province China; 2 Department of AI and IT, Second Affiliated Hospital, School of Medicine, Zhejiang University Hangzhou, Zhejiang Province China; 3 Department of Obstetrics, Second Affiliated Hospital of Zhejiang University Hangzhou, Zhejiang Province China; 4 Department of Obstetrics, Xinglin Hospital of Xiamen Xiamen, Fujian Province China; 5 School of Public Health, Hangzhou Medical College Hangzhou, Zhejiang Province China; 6 School of Nursing, Zhejiang Chinese Medical University Hangzhou, Zhejiang Province China; 7 NetEase ThunderFire UX User Experience Center, Netease Group Hangzhou, Zhejiang Province China; 8 School of Nursing, Southwest Medical University Luzhou, Sichuan Province China; 9 School of Medical Technology and Information Engineering, Zhejiang Chinese Medical University Hangzhou, Zhejiang Province China; 10 National Key Laboratory of Transvascular Implantable Devices, Second Affiliated Hospital, School of Medicine, Zhejiang University Hangzhou, Zhejiang Province China; 11 Intelligent Medical Research Center, Zhejiang University Institute of Computer Innovation Technology Hangzhou, Zhejiang Province China; 12 Clinical Research Center, Affiliated Hospital of Southwest Medical University Luzhou, Sichuan Province China; 13 School of Medical Information and Engineering, Southwest Medical University Luzhou, Sichuan Province China; 14 Center for Medical Informatics, Institute of Advanced Clinical Medicine, Peking University Beijing China

**Keywords:** pregnancy management app, user satisfaction, user-generated content, two-factor theory, latent Dirichlet allocation, LDA, topic model

## Abstract

**Background:**

China’s 3-child policy has increased the demand for scientific and personalized pregnancy health management. The convenience of mobile health has promoted the use of pregnancy management apps among pregnant women. User satisfaction has a significant impact on continued use intention. Systematically evaluating user satisfaction with pregnancy management apps is of great significance in promoting the digital transformation of maternal-infant health care.

**Objective:**

This study aimed to explore user satisfaction with pregnancy management apps by mining user-generated content and analyzing the differences in user satisfaction among different operating systems. Under the guidance of Herzberg two-factor theory, this study explored the demand structure in user experience and investigated the influencing factors on satisfaction from 2 aspects: user satisfaction and dissatisfaction, providing reference for the development of mobile health apps for pregnancy management.

**Methods:**

We screened pregnancy management apps from the app stores of 5 mobile phone manufacturers in mainland China and collected the app-based reviews and ratings posted by users. We performed topic clustering and semantic parsing using the latent Dirichlet allocation and DeepSeek-R1 models. Influencing factors for satisfaction and dissatisfaction were identified using the Tobit regression model. The Kano model was used to classify the factors as basic or attractive. The Wald test was applied to analyze the differences in the effects across various factors.

**Results:**

We examined 86 pregnancy management apps, amounting to 180,107 reviews in total. The overall satisfaction rate with pregnancy apps was relatively high (72.34%). Android users had higher satisfaction rates than iOS users (89.32% vs 60.75%). User reviews were clustered into 12 themes, categorized into 3 types: technical security support, basic service experience, and maternal-infant scenarios. The basic factors causing dissatisfaction included system login (β=2.829; *P*<.001) and privacy disclosure (β=1.955; *P*<.001). Attractive factors boosting satisfaction included storage optimization (β=0.220; *P*<.001), page design (β=0.223; *P*<.001), function provision (β=0.023; *P=*.001), platform feedback (β=0.222; *P*<.001), physicians’ inquiries (β=0.356; *P*<.001), menstrual management (β=0.209; *P*<.001), pregnancy guidelines (β=0.306; *P*<.001), parenting science popularization (β=0.238; *P*<.001), growth record (β=0.401; *P*<.001), and maternal-infant community (β=0.307; *P*<.001).

**Conclusions:**

There were significant differences in user satisfaction between the iOS and Android platforms, reflecting the heterogeneity of responses to user demands in different technological ecosystems. Twelve themes showed the multilevel needs of pregnant women for pregnancy management apps, forming a progressive service chain: security guarantee to demand satisfaction to experience improvement. Twelve factors had asymmetric correlations with user satisfaction and dissatisfaction with pregnancy management apps. Two factors were related to basic attributes of the apps and 10 factors to attractive attributes. By distinguishing between basic and attractive factors, the use of pregnancy management apps may be improved.

## Introduction

### Background

Childbirth is a significant event in a woman’s life course. Health during pregnancy is related to the safety of mothers and infants and the well-being of families. Against the backdrop of China’s 3-child policy [1], the proportion of older mothers increased and the birth interval shortened. Women’s demand for scientific and personalized health management during pregnancy has expanded. Under the traditional face-to-face medical consultation model, pregnant women usually seek medical services only after they encounter health problems. This offline pregnancy management model has significant limitations. On the one hand, in areas with scarce medical resources, the high cost of medical treatment may lead to the delay or neglect of prenatal checkups [2]. On the other hand, during natural disasters or infectious disease epidemics, the face-to-face consultation process may trigger the risk of cross infection and exacerbate the tightness of medical resources [3].

In contrast, the introduction of mobile health (mHealth) apps has significantly enhanced the accessibility and convenience of pregnancy management. These effectively promote the continuous participation of target groups, such as pregnant women, in daily maternal-infant health management [4]. As a special group with specific health needs, the dynamic changes in the physical and mental states of pregnant and postpartum women pose higher-dimensional requirements for digital health services. The satisfaction of pregnant women directly affects their intention to continue using mHealth [5]. Systematically evaluating user satisfaction with pregnancy management apps and its driving factors is an urgent issue for optimizing digital health services, holding strategic significance for promoting the digital transformation of maternal and child health care.

User-generated content (UGC) refers to the diversified information forms that are actively created, published, and disseminated by users through digital media [6]. Research on exploring user satisfaction with mHealth mainly focused on chronic disease management [7,8]. The research on pregnancy management apps was not sufficient, with limited research subjects, such as only studying the most popular apps [9], or focusing only on certain functions, such as physical exercise and nutritional matching. [10,11]. The current research on exploring user satisfaction with mHealth apps has several flaws. First, single data sources have been used; previous studies mostly used survey-based methods to obtain users’ opinions on apps [12], which does not provide users’ spontaneous and unstructured insights. Second, fixed analytical dimensions have been used; previous studies lacked a professional management guidance framework to explore user satisfaction [13,14]. The two-factor theory in management proposed by Herzberg et al [15] suggests that the factors influencing user satisfaction can be categorized into attractive factors that enhance satisfaction and basic factors that mitigate dissatisfaction. Third, past studies have used technical methods that lack novelty, and their qualitative analyses have not been based on new technologies, such as large language models (LLMs). Using methods such as a general inductive content analysis approach may lead to accuracy bias or insufficient depth in the analysis [16].

### Objectives

We aimed to conduct topic clustering and qualitative analysis of user reviews using latent Dirichlet allocation (LDA) and LLMs and deeply explore the influencing factors of user satisfaction with pregnancy management apps under the guidance of the two-factor theory. Methodologically, we tested the applicability of LLMs in health informatics. Theoretically, we conducted a differentiated exploration of the influencing factors of user satisfaction with pregnancy management apps. Practically, this study can improve the development of digital health apps for pregnancy management by mining UGC.

## Methods

### Overview

**Figure 1:**
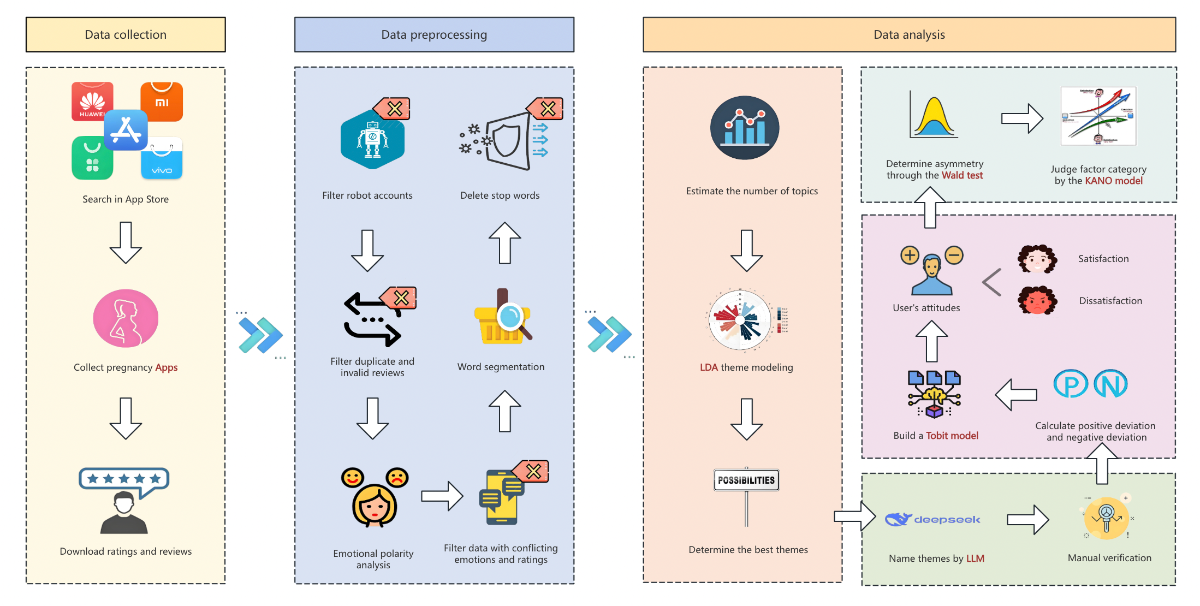
Analysis framework for evaluating user satisfaction with pregnancy management apps. In the data collection stage, we included apps that met the inclusion and exclusion criteria and collected user reviews; in the data preprocessing stage, we cleaned user reviews and performed sentiment analysis and segmentation; and in the data analysis stage, we used the latent Dirichlet allocation (LDA) and DeepSeek-R1 models to cluster and name topics, respectively, explored the influencing factors of user satisfaction through Tobit model, and discriminated the factors based on Kano model. LLM: large language model.

In this cross-sectional study, we included women in mainland China who were either preparing for pregnancy or already pregnant as research participants. All analysis code was written in Python (version 3.11.3; Python Software Foundation) and R (version 4.2.3; R Foundation for Statistical Computing). Data analysis was completed in Stata (version 18.0; StataCorp LLC). The technical road map is shown in Figure 1.

### Data Collection

#### App Filtering

**Figure 2:**
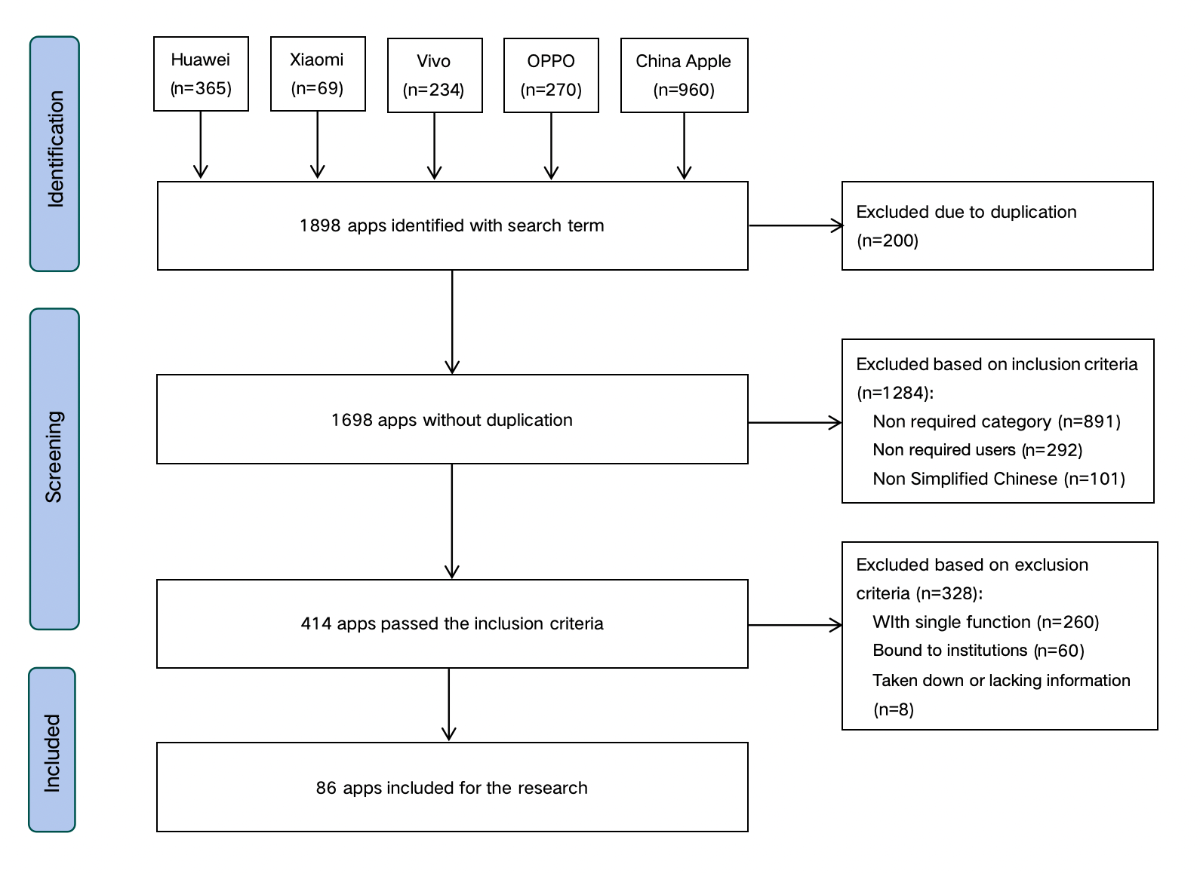
Screening flowchart of pregnancy management apps.

We selected the mainstream mobile operating systems of mainland China, namely iOS and Android. We chose the app stores of Apple, Huawei, Xiaomi, Vivo, and OPPO, which are the top 5 mobile phone manufacturers in terms of monthly activity ranking [17]. These official app stores are the first choice for users to download apps. In February 2025, we conducted a search in the app stores using the keywords “pregnancy” or “pregnancy period” or “pregnant woman” or “preparing for pregnancy” or “gestation.” The inclusion and exclusion criteria are shown in Textbox 1. Two researchers conducted independent screening based on the inclusion and exclusion criteria (MX and YH), and they received standard training before screening. We reviewed the consistency of screening between 2 researchers by calculating the κ value. Refer to Figure 2 for the screening process. Any differences between the 2 researchers were arbitrated and resolved by an obstetrician (LJ). The screening process followed the PRISMA guidelines.

**Inclusion and exclusion criteria for pregnancy management apps.**

Inclusion criteria

1. The detection results with the search terms “pregnancy” or “pregnancy period” or “pregnant woman” or “preconception” or “gestation”
2. The category of the app belongs to health, medical care, and maternal-infant care (priority should be given to app information; otherwise, it came from the researcher’s judgment)
3. The target users are mainly those who are preparing for pregnancy naturally, pregnant women, and new mothers
4. App description written in simplified Chinese

Exclusion criteria

1. Apps only for menstrual tracking, calculating gestational age or due date, pregnancy exercise, pregnancy music, weight and diet control during pregnancy, baby name search, baby photo editing, baby monitoring, and purchasing baby products
2. Apps used by members and patients related to special programs (such as fitness centers, clubs, or other paid memberships) or health care facilities (such as clinics or hospitals)
3. As of the search date, the app has been removed from the app store or has no platform rating in the app store

#### Collection of User Reviews

Using Qimai, a mobile app analysis platform, we obtained the user reviews of the apps. Considering the impact of the COVID-19 pandemic and China’s 3-child policy on the increased demand for online pregnancy management, we set the search scope from January 1, 2019, to December 31, 2024, including data before and after the COVID-19 pandemic and the implementation of China’s 3-child policy. All the aforementioned 5 app stores contain users’ ratings and reviews. The rating range was from 1 to 5 points. The reviews were self-published by users.

### Data Preprocessing

**Figure 3:**
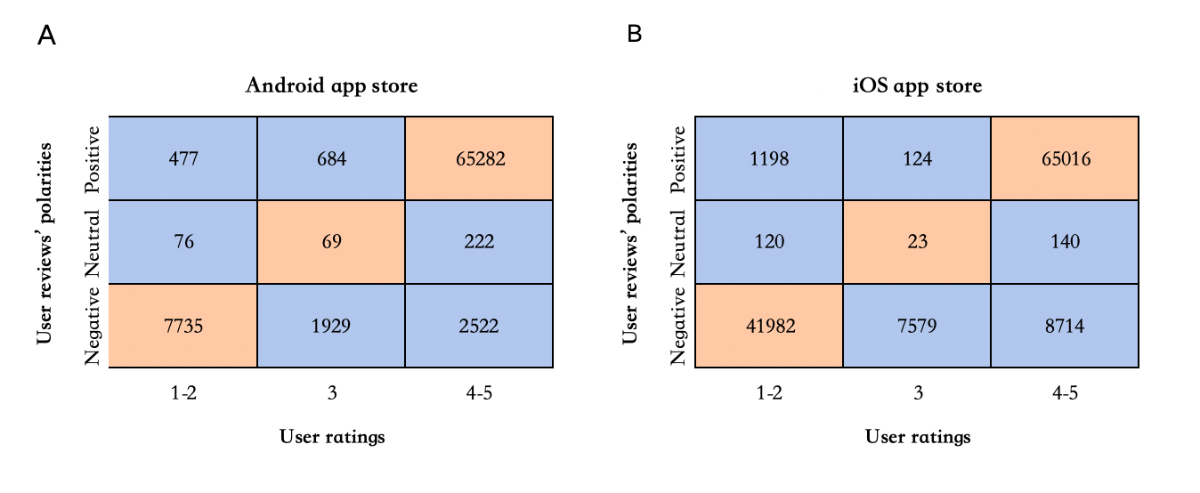
User sentiment polarity analysis of pregnancy management apps. (A) and (B) show the user reviews’ polarities and user ratings in the Android app stores and the Apple App Store, respectively. The user reviews in the orange cells were retained because they represented user ratings that aligned with user sentiment. The user reviews in the blue cell were deleted because they represented a conflict between user ratings and user emotions.

First, we used the *tweetbotornot* package in the R programming language to delete bot-created reviews and eliminate duplicate, blank, and invalid reviews. Second, we conducted sentiment analysis using the sentiment knowledge enhancement pretraining algorithm and deleted data where the sentiment polarity in the reviews contradicted the ratings [18]. The sentiment analysis of user reviews is shown in Figure 3. Finally, through the Jieba Chinese word segmentation dictionary in Python, we split the Chinese sequences into independent words. We deleted the stop words in user reviews according to the comprehensive stop word list aggregated from Baidu (Harbin Institute of Technology) and the Chinese stop word list [19].

### Data Analysis

#### LDA for Topic Modeling

LDA is a text mining method based on probabilistic generative models that is widely used in the analysis of UGC (Figure S1 in Multimedia Appendix 1) [20]. We used LDA for topic modeling. First, we determined the number of topics through perplexity. The lower the perplexity, the better the corresponding model [21]. The position where the decline of the perplexity curve slows down is taken as the optimal number of topics. Second, we generated the clustering topics. Using the Gensim software package in Python, we constructed a topic clustering model for user reviews. Third, we determined the best theme. The LDA generated the distribution probabilities of user reviews on different topics [22]. The topic with the highest distribution probability was set as the best topic.

#### DeepSeek-R1 for Topic Naming

We provided topic naming and descriptive labels for each cluster through LLMs. This method has been proven to be superior to pure humans or pure LLMs in qualitative analysis [23]. Prompt words were input to DeepSeek-R1 with 30 keywords of each cluster, and topic names and descriptive labels were output for 12 clusters. On the basis of the names and main keywords of clustering topics, we used LLM to classify the topics. The prompt words are shown in Table S1 in Multimedia Appendix 1. This process was supervised and verified by 2 researchers (MZ and PX) [24]. They used a 1 to 5 Likert scale to score relevant indicators, specifically including 3 indicators: accuracy of naming, interpretability of description, and rationality of classification, with a total of 6 items. The verification results are shown in Table S2 in Multimedia Appendix 1.

#### Tobit Model for Exploring the Relevant Factors

According to the two-factor theory, we introduced 2 dependent variables: positive deviation (PD) and negative deviation (ND), to measure user satisfaction and user dissatisfaction, respectively [25]. The 2 variables were defined as the absolute values of the differences between the user rating and the app’s comprehensive rating in the app stores. The value range of the PD and ND was 0 to 4. For the selection of independent variables, LDA outputs the probability distribution of each user review in a certain topic, reflecting the attention of different users to each topic. We defined attention as the independent variable. After normalizing the topic probability, the data range was compressed to 0 to 1 [26].

The Tobit model is widely used for mining user comment satisfaction [25,27]. Given that user ratings for the apps were bounded between 0 and 5, the dependent variables PD and ND exhibited censored data characteristics. Ordinary least squares regression may result in biased parameter estimates by treating censored boundary values as true measurements [28]. In contrast, the Tobit model can incorporate all observed outcomes to estimate the regression line through maximum likelihood [29]. Therefore, we used the Tobit model to analyze the influencing factors of user satisfaction. The regression models are shown in equations 1 and 2. In addition, we conducted a difference test on the coefficients of the PD and ND using the Wald test to evaluate asymmetry [25]. The significance was set as bilateral *P*<.05.

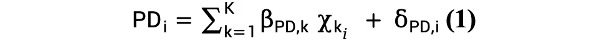

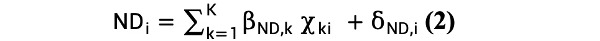





β_k_ refers to the correlation coefficient between the kth topic χ_ki_ of review i and user satisfaction PD_i_ and dissatisfaction ND_i_, k is the number of topics, i is the number of user reviews, and δ_i_ is the error term.

#### Kano Model for Attribute Division

The Kano model is derived from the two-factor theory and widely used in analyzing user needs and behaviors. It categorizes product attributes into 5 types: attractive factors, basic factors, expected factors, undifferentiated factors, and inverse factors [30]. We classified the influencing factors by comparing their degrees of influence on user satisfaction and dissatisfaction. Referring to the discrimination criteria of previous studies, 6 discrimination indicators were set (Figure S2 in Multimedia Appendix 1) [25].

### Ethical Considerations

All data used in this study were sourced from publicly accessible websites, and all user information was anonymized. Sensitive data were not collected for this study, and no compensation was provided for participants. All data relevant to this study were stored on a password-encrypted computer, and only the researchers had access to the data. Ethics approval was not required according to the ethical review by the ethics committee of the Second Affiliated Hospital Zhejiang University School of Medicine [31].

## Results

### Descriptive Statistics of the Pregnancy Management Apps

**Figure 4:**
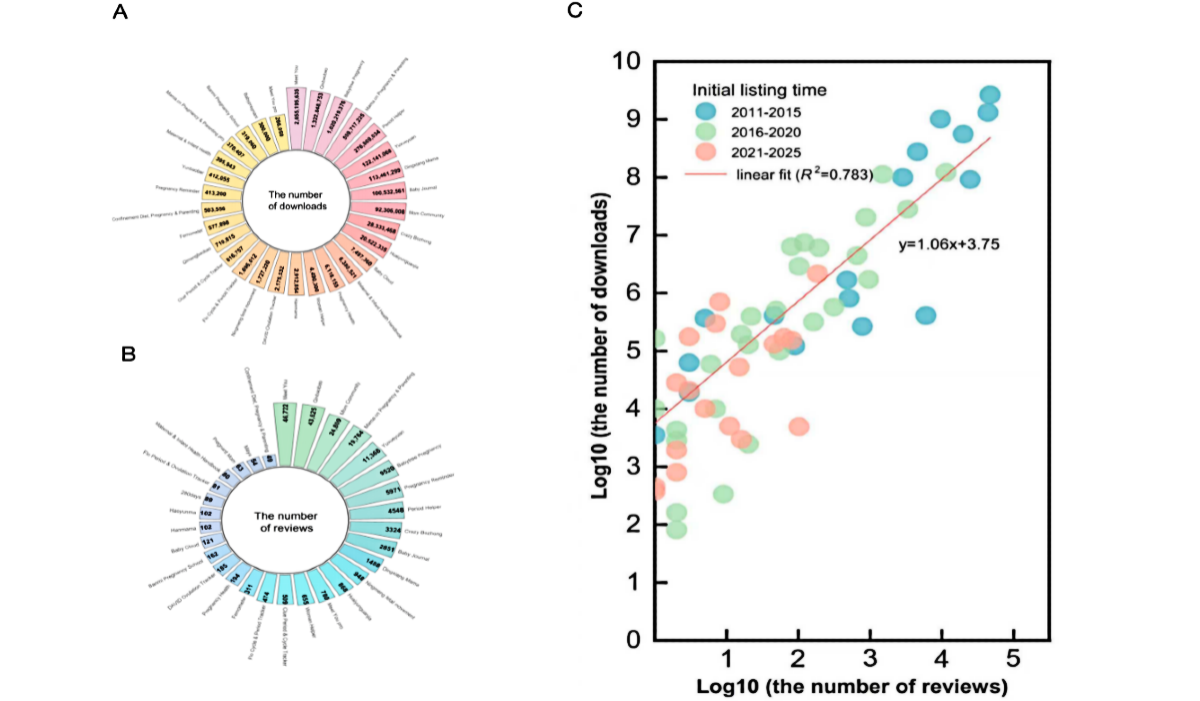
Distribution and correlation of downloads and reviews of pregnancy management apps. (A) The number of user downloads of the top 30 apps; (B) the number of user reviews of the top 30 apps; (C) the relationship between app downloads and reviews. The number of app downloads and reviews in this chart have been converted to log scale.

**Figure 5:**
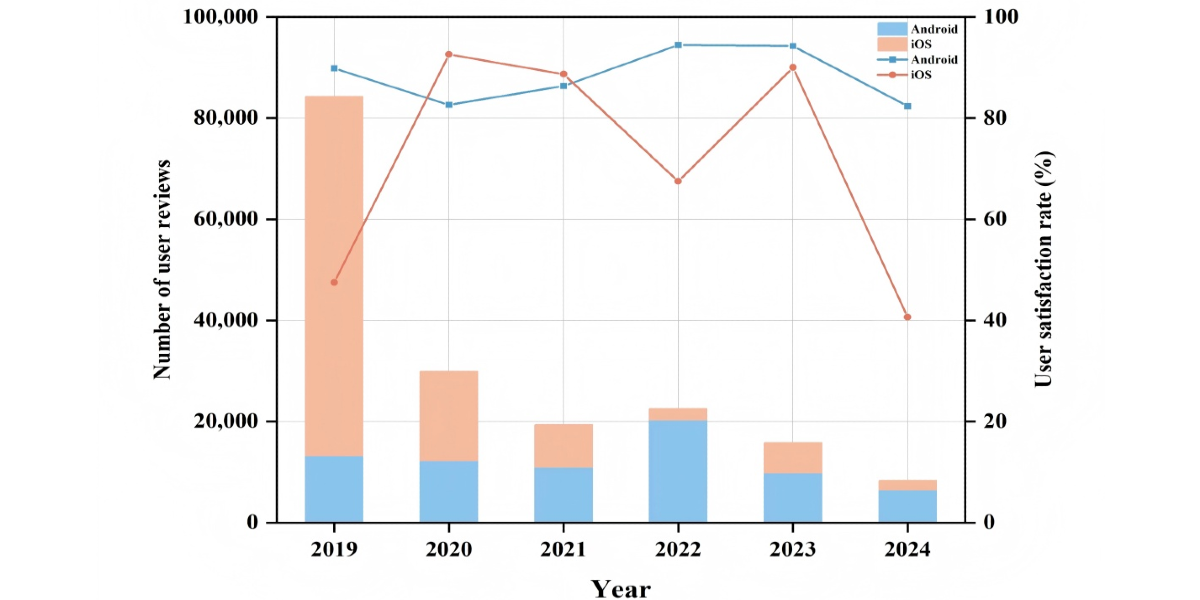
Number and satisfaction of user reviews on the pregnancy management apps. The bar chart represents the number of user reviews, corresponding to the y-axis on the left. The line graph represents the user satisfaction rate, corresponding to the y-axis on the right.

We searched a total of 1698 apps and ultimately included 86 (5.06%) pregnancy management apps with 180,107 reviews. The κ value (0.86) indicated that the screening processes of the 2 researchers were highly consistent. Figure 4 shows the distribution and correlation of downloads and reviews of the apps. The pregnancy management app market presented a certain degree of monopoly. In terms of download volume, the top 10 apps accounted for 99.05% of the total download volume (mean 102 million, SD 397 million), while in terms of review volume, the top 10 apps contributed to 95.78% of the total comment volume (mean 2904.95, SD 8935.12). The relationship between user reviews and downloads followed a power-law distribution. More information about downloads and reviews of apps is provided in Figure S3 in Multimedia Appendix 1. The ratio of user reviews on iOS and Android systems was approximately 3:2, reaching its peak in 2019 and 2022, respectively, which may be related to the catalytic effect of the COVID-19 pandemic on digital health apps [32]. In total, 72.34% (130,298/180,107) of the ratings reached ≥4 stars, with a satisfaction rate of 72.34%. The satisfaction rates of the iOS system and the Android system were 60.75% and 89.32%, respectively. The satisfaction rates of Android users were relatively stable, staying between 80% and 95% from 2019 to 2024, while the satisfaction rates of iOS users fluctuated significantly, with a substantial decline in both 2022 and 2024 (Figure 5).

### Distribution of User Review Topics for the Pregnancy Management Apps

**Figure 6:**
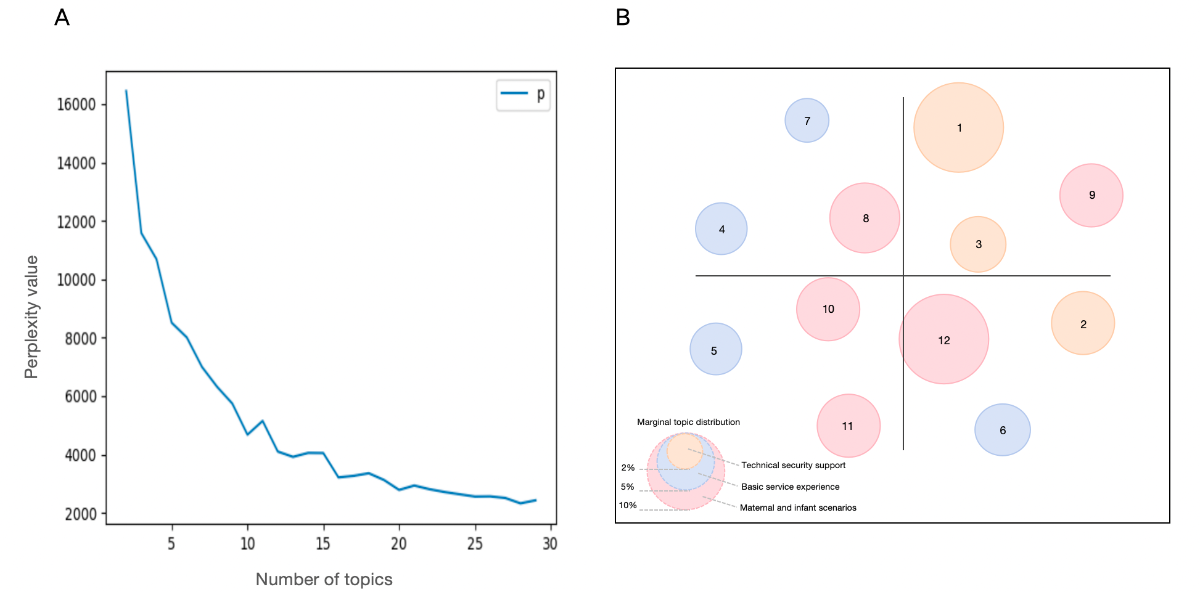
Perplexity curve and topic distribution for user reviews of pregnancy management apps. (A) Perplexity curve for latent Dirichlet allocation (LDA) analysis of user reviews of pregnancy management apps. The optimal number of themes is generally located at the inflection point where the curve transitions from a steep drop to a gentle one. (B) Topic clustering effect in LDA analysis of user reviews of pregnancy management apps. Circles correspond to clustering themes. The numbers in the circles correspond to the theme number, while the colors of the circles represent the categories of the theme.

On the basis of the perplexity curve of the user review clustering, we preliminarily estimated that the optimal number of themes was approximately 12 to 15. Combined with the actual clustering situation of LDA, we confirmed that when the number of topics was 12, the clustering effect was the best. The perplexity curve and theme clustering effect are shown in Figure 6. The clustering themes included system login, privacy disclosure, storage optimization, page design, function provision, platform feedback, physicians’ inquiries, menstrual management, pregnancy guidelines, parenting science popularization, growth record, and maternal-infant community. The names, key words, and descriptions of the clustering topics are shown in Table 1.

**Table 1:** Basic information on clustering topics of the user reviews on the pregnancy management apps (N=180,107).

Category and themes		Keywords	Description	Corpus distribution, n (%)	Quantity of reviews, n (%)
**Technical security support**					
	System login	“login,” “withdrawal,” “system,” “network,” “bind account”	Problems such as sudden exit, lag, and updates when logging into the software	227,092 (16.29)	45,520 (25.27)
	Privacy disclosure	“information,” “leakage,” “advertisement,” “telemarketing”	User information is leaked, and advertisements are forced to be pushed	109,434 (7.85)	7979 (4.43)
	Storage optimization	“photos,” “upload,” “version,” “automatic,” “update”	Cloud storage of generated content (such as baby photos)	82,668 (5.93)	11,481 (6.37)
**Basic service experience**					
	Page design	“search,” “support,” “interface,” “dazzling”	The fonts, styles, etc displayed on the interface	68,588 (4.92)	11,633 (6.46)
	Function provision	“diary,” “functions,” “completeness,” “games,” “homepage”	Functions related to preconception and pregnancy management	67,751 (4.86)	3429 (1.9)
	Platform feedback	“debunking rumors,” “replying,” “customer service,” “feedback,” “information”	The official platform provides feedback on users’ inquiries	84,062 (6.03)	23,239 (12.9)
	Physicians’ inquiries	“doctor,” “expert,” “guide,” “tell,” “answer”	Physicians or experts provide detailed answers to users’ questions	53,114 (3.81)	9478 (5.26)
**Maternal-infant scenarios**					
	Menstrual management	“menstruation,” “menstrual period,” “reminders,” “predictions,” “very accurate”	The use of functions, such as female menstrual cycle recording, prediction, and health reminders	150,001 (10.76)	10,420 (5.79)
	Pregnancy guidelines	“pregnancy,” “preconception,” “prenatal checkups,” “diet,” “expectant mothers”	Knowledge acquisition and prenatal checkup management tools for each stage of pregnancy	110,828 (7.95)	8117 (4.51)
	Parenting science popularization	“parenting,” “practicality,” “methods,” “knowledge,” “learning”	Learning and sharing of authoritative content on infant and toddler care knowledge	121,562 (8.72)	10,515 (5.84)
	Growth record	“growth,” “baby,” “memory,” “time,” “moment”	Graphic and textual records and memory storage of children’s development milestones	120,028 (8.61)	15,115 (8.39)
	Maternal-infant community	“mother and baby,” “community,” “experience,” “companionship,” “selling goods”	Experience exchange and emotional support interaction among pregnant and postpartum users	198,932 (14.27)	23,181 (12.87)

These 12 themes were summarized into 3 major categories: technical security support, basic service experience, and maternal-infant scenarios. Technical security support included 3 core dimensions: system login, privacy disclosure, and storage optimization. From the stability of user authentication and compliance with the protection of data to the efficient management of storage resources, they jointly built the reliability of the underlying technical foundation of the platform. The basic service experience involved 4 key elements: page design, function provision, platform feedback, and physicians’ inquiries. These formed the basic service framework of health-related apps and directly affected users’ judgment on the usability of the product. Maternal-infant scenarios integrated the full-cycle needs of users from preconception to parenting, covering 5 core scenarios: menstrual management, pregnancy guidelines, parenting science popularization, growth records, and maternal-infant communities, building a continuous service ecosystem for the user’s life cycle.

### Factors Related to User Satisfaction With the Pregnancy Management Apps

We used the variance inflation factor to test the multicollinearity among variables. The results showed that the variance inflation factors of all independent variables were less than 5, indicating that there was no serious multicollinearity problem (Table S3 in Multimedia Appendix 1) [33]. Models 1 and 2 represent the influence of different factors on satisfaction and dissatisfaction, respectively. The results in Table 2 showed that the 12 factors had a significant impact on both user satisfaction and dissatisfaction. System login and privacy disclosure had a positive impact on user dissatisfaction, indicating that the more users discussed system login and privacy disclosure, the more they showed dissatisfaction. The other 10 factors had a positive impact on user satisfaction, indicating that the more users discussed the aforementioned 10 factors, the more satisfaction they showed. The Wald test showed that the influences of the 12 factors on user satisfaction and dissatisfaction were asymmetric. Among them, the system login (χ^2^_1_=16,186.3) had the strongest asymmetry of satisfaction and dissatisfaction with users (Table S4 in Multimedia Appendix 1).

**Table 2:** Factors influencing user satisfaction with pregnancy management apps.

Factors	Model 1^a^, β (SE; 95% CI)	Model 2^a^, β (SE; 95% CI)
Theme 1: system login	−0.474^b^ (0.005; −0.484 to −0.464)	2.829^b^ (0.023; 2.784 to 2.875)
Theme 2: privacy disclosure	−0.232^b^ (0.007; −0.247 to −0.218)	1.955^b^ (0.027; 1.902 to 2.008)
Theme 3: storage optimization	0.220^b^ (0.005; 0.211 to 0.229)	−2.796^b^ (0.042; −2.878 to −2.713)
Theme 4: page design	0.223^b^ (0.005; 0.212 to 0.233)	−2.032^b^ (0.036; −2.103 to −1.962)
Theme 5: function provision	0.023^c^ (0.007; 0.009 to 0.038)	−0.420^b^ (0.040; 0.340 to 0.499)
Theme 6: platform feedback	0.222^b^ (0.004; 0.214 to 0.230)	−3.587^b^ (0.040; −3.665 to −3.510)
Theme 7: physicians’ inquiries	0.356^b^ (0.005; 0.346 to 0.365)	−3.665^b^ (0.042; −3.747 to −3.583)
Theme 8: menstrual management	0.209^b^ (0.006; 0.196 to 0.221)	−0.501^b^ (0.029; 0.445 to 0.557)
Theme 9: pregnancy guidelines	0.306^b^ (0.006; 0.295 to 0.318)	−2.570^b^ (0.046; −2.659 to −2.481)
Theme 10: parenting science popularization	0.238^b^ (0.006; 0.227 to 0.249)	−1.168^b^ (0.036; −1.237 to −1.098)
Theme 11: growth record	0.401^b^ (0.005; 0.391 to 0.410)	−3.109^b^ (0.042; −3.192 to 3.026)
Theme 12: maternal-infant community	0.307^b^ (0.004; 0.298 to 0.315)	−2.416^b^ (0.028; −2.472 to −2.360)

^a^Models 1 and 2 represent the influence of different factors on satisfaction and dissatisfaction.

^b^*P=* <.001.

^c^*P=*.001.

### Attribute Distribution of the Relevant Factors for the Pregnancy Management Apps

**Figure 7:**
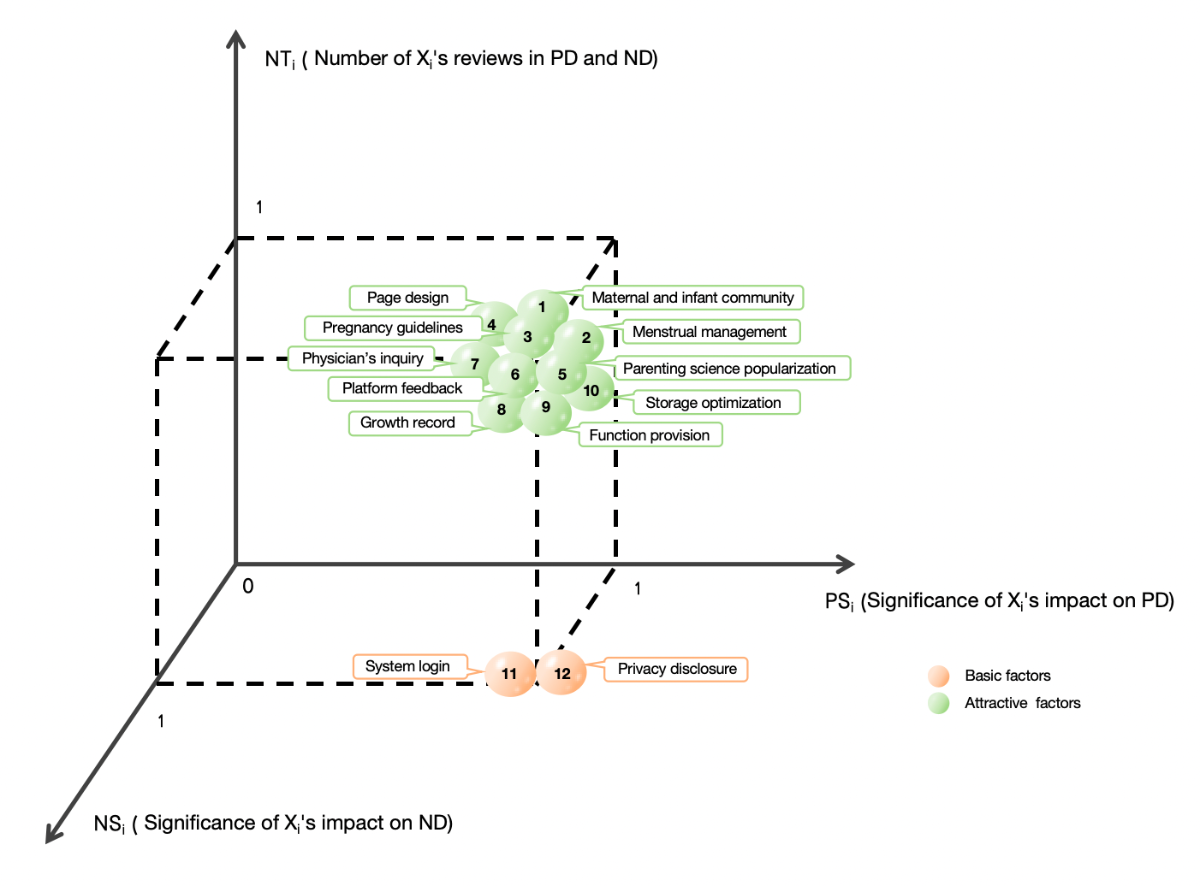
Attribute distribution of factors affecting the user satisfaction rate of pregnancy management apps.

We classified the 12 influencing factors into 2 basic factors and 10 attractive factors (Table S5 in Multimedia Appendix 1). System login and privacy disclosure were basic factors. These factors had significant impacts on both user satisfaction and dissatisfaction, with significant differences in the influence coefficients, and the number of dissatisfied reviews was greater than that of satisfied reviews (PS_i_=1, NS_i_=1, CD_i_=1, and NT_i_=0). The other 10 factors were attractive factors. These factors had significant influences on both user satisfaction and dissatisfaction. There were significant differences in the influence coefficients, and the number of satisfied reviews was greater than that of dissatisfied reviews (PS_i_=1, NS_i_=1, CD_i_=1, and NT_i_=1). The distributions of different factors are shown in Figure 7.

## Discussion

### Principal Findings

In this study, we focused on pregnant women or those preparing for pregnancy, explored the user downloads and reviews of pregnancy management apps, and analyzed the user satisfaction and influencing factors. The main findings of this study were as follows: first, the overall satisfaction rate of pregnancy apps was relatively high (72.34%), with certain system differences. Android users had higher satisfaction rates than iOS users (89.32% vs 60.75%), which may be related to differences in user demand stratification and technology ecosystems. Second, the topics of user reviews on pregnancy management apps can be divided into 3 categories: technical safety support, basic service experience, and maternal-infant scenarios. These factors had an asymmetric impact on user satisfaction and dissatisfaction. Basic factors causing dissatisfaction included system login (β=2.829; *P*<.001) and privacy disclosure (β=1.955; *P*<.001). Attractive factors boosting satisfaction included storage optimization (β=0.220; *P*<.001), page design (β=0.223; *P*<.001), function provision (β=0.023; *P=*.001), platform feedback (β=0.222; *P*<.001), physicians’ inquiries (β=0.356; *P*<.001), menstrual management (β=0.209; *P*<.001), pregnancy guidelines (β=0.306; *P*<.001), parenting science popularization (β=0.238; *P*<.001), growth record (β=0.401; *P*<.001), and maternal-infant community (β=0.307; *P*<.001).

### Comparison to Previous Work

As far as we know, this is the first study to use LDA and DeepSeek-R1 for deep mining of UGC in pregnancy management apps. In theory, the study revealed the hierarchical structure of user experience in pregnancy management apps and verified the applicability of the two-factor theory in pregnancy health. In the vertical field of mHealth apps, the technical fault tolerance of basic factors is significantly lower than that of traditional consumer apps. Meanwhile, the dimensions of attractive factors exhibit significant service chain characteristics. In practice, the research results provided a reference for relevant entities of pregnancy management apps. Suppliers need to build a comprehensive service ecosystem throughout the process to achieve sustained growth in user satisfaction. The government should further promote the development of online health care, pay attention to the health needs of women of childbearing age, and promote the realization of medical equity.

First, the user satisfaction rate of the pregnancy management apps in this study was 72.34%, roughly the same as those reported in a systematic review of the maternal health app (77.6%) [34]. This proportion was higher than other health apps, such as mental health (65.09%) [35]. This may be because women’s health apps excel in stability, compatibility, privacy, and other aspects [36]. In terms of user satisfaction for different mobile operating systems, we found that the satisfaction level of Android users was higher than that of iOS users. The differences in user satisfaction may come from the distribution strategies between the 2 systems and the stratification of user groups. On the one hand, the Android system adopts a hierarchical review system for app updates, while the unified review standards of the Apple App Store may lead to a lag in app feature iteration. On the other hand, the prices of Apple iPhones are generally higher than those of Android phones in China, so iOS users may have more stringent requirements for refined features, such as device collaboration and data accuracy. The result was roughly consistent with the user ratings of the maternal and infant health apps [37], contrary to the user ratings of breastfeeding apps [38]. This might be because the breastfeeding apps are driven by a tracking function. The accuracy of iPhones is higher.

Second, we categorized the topics of user reviews on pregnancy management apps into technical safety support, basic service experience, and maternal-infant scenarios, forming a progressive service chain: security guarantee to demand satisfaction to experience improvement. Previous studies lacked the classification and generalization of user reviews on pregnancy management. In contrast, our research classified the topics as a chain combination [39,40]. We proposed that technical security support constituted the underlying guarantee. The absence of system stability and privacy data protection mechanisms may undermine the user trust foundation, leading to a decrease in users’ willingness to continue using the app. The basic service experience focused on the basic needs of users, reflecting their overall perception and expectations of the quality of basic product services. Maternal-infant service scenarios ran through an entire life cycle: preconception to pregnancy to postpartum, demonstrating significant stage progression characteristics of user needs. Among them, menstrual management provided physiological cycle prediction for the preconception stage, pregnancy guidelines met the scientific management needs during pregnancy, parenting science popularization and growth record served the postpartum care of infants and toddlers, and the maternal-infant community strengthened users’ sense of belonging through emotional resonance.

Third, we validated the asymmetry between these influencing factors of user satisfaction and dissatisfaction, which was an innovation compared to previous research. Previous research lacked exploration into the differences between factors that contributed to user satisfaction and dissatisfaction [41,42]. Our research categorized the influencing factors into basic factors and attractive factors, emphasizing the need for manufacturers to treat the 2 factors differently. The 2 basic factors of system login and privacy disclosure had a significant promoting effect on user dissatisfaction. Compared to previous studies, this promoting effect was greater [8,43]. This may be related to the particularity of pregnant women. Specifically, pregnant women’s demand for real-time monitoring and other data records is more rigid, so they are more likely to be dissatisfied with the occurrence of negative events, such as app flashback or advertising promotion. Attractive factors, such as physicians’ inquiries, had a significant promoting effect on user satisfaction, aligning with established empirical evidence in extant literature [44]. It is recommended that suppliers establish more convenient communication channels between physicians and users and ensure the professionalism and responsiveness of doctors.

### Limitations

There were some limitations in this study. First, the app stores where user comments are sourced may not be comprehensive enough. We selected only the official app stores of mobile phone manufacturers as the data source. Tencent, Qihoo, and Baidu have been increasingly marginalized by manufacturers’ app stores in recent years, while Google Play services are not authorized in mainland China and thus are not available without a VPN (virtual private network) circumvention. We did not choose these third-party app stores as data sources. Second, sample self-selection bias may affect the conclusion validity. Users with high satisfaction lack the motivation to actively publish reviews, which may lead to an overestimation of users’ dissatisfaction. However, focusing on the user population who actually use and post comments was in line with the clinical study design principles of the per protocol set. Third, there were certain limitations in the language processing of user reviews. Those consisting solely of emojis or punctuation marks were deleted due to the difficulty in conducting topic clustering. It may lead to a loss of some semantics. Furthermore, this research lacked user reviews from other countries. Finally, there was a lack of dynamic analysis of app user reviews. User focus may change with the development stage of the app.

### Future Directions

In future research, we intend to explore the longitudinal trend of user reviews on pregnancy management apps over time and expand cross-border analysis of user comments on these apps, such as the United States. Our research advocated that suppliers should prioritize strengthening the bottom line of health care and reconstruct the service ecosystem with the functional improvement of the maternal-infant scenarios as the core. Medical institutions should rely on medical and health technology innovation to expand the accessibility of high-quality maternity and childbirth services, bridge regional health disparities, and ensure service fairness. Simultaneously, they should identify the individualized needs of pregnant women and achieve precise intervention in pregnancy management through intelligent tools. Regulators should strengthen the approval of digital health apps and raise the threshold for app market entry, such as including system stability requirements.

### Conclusions

On the basis of the two-factor theoretical framework, we conducted LDA topic modeling and Tobit regression analysis on 180,107 user reviews from 86 pregnancy management apps, systematically revealing the demand structure and satisfaction influencing factors of pregnant woman for mobile health services. The research found that user reviews can be clustered into 3 major dimensions: technical security support, basic service experience, and maternal-infant scenarios. Among them, system login and privacy disclosure, as basic factors, had a decisive impact on user dissatisfaction, while 10 other influencing factors, such as physicians’ inquiries, served as attractive factors to significantly enhance satisfaction. In addition, there were significant differences in user satisfaction between the iOS and Android platforms, reflecting the heterogeneity of responses to user demands in different technological ecosystems. These findings provided empirical evidence for understanding the user experience of pregnancy management apps and revealed the internal mechanism of satisfaction formation in medical and health mobile apps.

## Appendix

Multimedia Appendix 1 Supplementary tables and figures.

## Abbreviations

LDA: latent Dirichlet allocation
LLM: large language model
mHealth: mobile health
ND: negative deviation
PD: positive deviation
UGC: user-generated content
VPN: virtual private network

## Data Availability

We used the official website of Qimai apps for data collection [45]. The source code for our analysis is publicly available on GitHub [46].
