# Supplementary material for: User Satisfaction With Pregnancy Management Apps in Mainland China: User-Generated Content Analysis and Text Mining Study
Source: J Med Internet Res. 2025 Aug 21;27:e78828. doi: 10.2196/78828 (PMC12411797; doi:10.2196/78828)
Supplement: Multimedia Appendix 1 [file jmir_v27i1e78828_app1.docx]

# Attachment

# Supplementary Table 1 Prompt words for topic naming and description input

| **Steps** | **Prompt words** | **Input data** |
| --- | --- | --- |
| Theme naming | You are an expert in obstetrics and gynecology and are now conducting a study on the user satisfaction of a pregnancy management Apps. After clustering the online user reviews by topic using LDA, 12 related topics were generated, and each topic had 30 main keywords. Please name these 12 topics respectively, and each topic name should be within 6 characters. | The 30 main keywords corresponding to the 12 themes respectively |
| Theme description | Please interpret the 12 topics named above. Describe the content of each topic in one sentence respectively. The sentences should be easy to understand and accurately summarize the content of the topic. | The names of 12 themes and their corresponding 30 main keywords |
| Theme classification | Please categorize the 12 themes based on their commonalities, with approximately 3 to 5 categories. Name each major category and provide explanations. | The names of 12 themes and their corresponding 30 main keywords |

# Supplementary Table 2 Manually supervise evaluation on the use of LLM

| **Serial number** | **Indicator information** | | **Score** | | | | | **Expert scoring** | |
| --- | --- | --- | --- | --- | --- | --- | --- | --- | --- |
|  | **Evaluation index** | **Meaning explanation** | **1** | **2** | **3** | **4** | **5** | **Expert 1** | **Expert 2** |
| 1 | Accuracy of naming | The topic name can fully cover the core keywords of the topic | Completely disagree | Disagree | Neutral | Agree | Completely agree | 5 | 5 |
|  |  | The topic name can precisely summarize the core semantics of the topic | Completely disagree | Disagree | Neutral | Agree | Completely agree | 4 | 4 |
| 2 | Interpretability of description | The theme description is clear and easy to understand | Completely disagree | Disagree | Neutral | Agree | Completely agree | 5 | 5 |
|  |  | There is no ambiguity in the topic description | Completely disagree | Disagree | Neutral | Agree | Completely agree | 5 | 5 |
| 3 | Rationality of classification | The parent theme can reflect the common characteristics of the child themes | Completely disagree | Disagree | Neutral | Agree | Completely agree | 4 | 5 |
|  |  | The name of the parent topic can encompass the core semantics of all the child topics | Completely disagree | Disagree | Neutral | Agree | Completely agree | 5 | 5 |

Note:

Expert evaluation consistency = Number of consistent items/Total number of items = 5/6 =83.3%

Expert 1 recognition = Self-assessment score/Total evaluation score =28/30=93.3%

Expert 2 recognition = Self-assessment score/Total evaluation score =29/30=96.6%

# Supplementary Table 3 Multicollinearity tests for different factors

|  |  | **Variance inflation factor** | |
| --- | --- | --- | --- |
| **Serial number** | **Factor** | **Model 1** | **Model 2** |
| 1 | System login | 1.25 | 1.25 |
| 2 | Privacy disclosure | 1.03 | 1.03 |
| 3 | Storage optimization | 1.04 | 1.04 |
| 4 | Page design | 1.06 | 1.06 |
| 5 | Function provision | 1.00 | 1.00 |
| 6 | Platform feedback | 1.14 | 1.14 |
| 7 | Physician's inquiry | 1.10 | 1.10 |
| 8 | Menstrual management | 1.02 | 1.02 |
| 9 | Pregnancy guidelines | 1.02 | 1.02 |
| 10 | Parenting science popularization | 1.02 | 1.02 |
| 11 | Growth record | 1.04 | 1.04 |
| 12 | Maternal-infant community | 1.05 | 1.05 |

# Supplementary Table 4 Coefficient difference test of user satisfaction and user dissatisfaction

| **Factors** | **χ2** | **p** | **Reviews in PD(%)** | **Reviews in ND(%)** |
| --- | --- | --- | --- | --- |
| Theme 1: system login | 16186.3 | <.001 | 13,828(31.43%) | 30,163(68.57%) |
| Theme 2: privacy disclosure | 4680.7 | <.001 | 2,432(30.94%) | 5,429(69.06%) |
| Theme 3: storage optimization | 4667.1 | <.001 | 10,634(95.18%) | 538(4.82%) |
| Theme 4: page design | 3446.5 | <.001 | 10,189(89.91%) | 1,144(10.09%) |
| Theme 5: function provision | 77.5 | <.001 | 2,004(60.13%) | 1,329(39.87%) |
| Theme 6: platform feedback | 8604.3 | <.001 | 22,234(96.54%) | 796(3.46%) |
| Theme 7: physician’s inquiry | 8383.4 | <.001 | 8,249(93.05%) | 616(6.95%) |
| Theme 8: menstrual management | 80.1 | <.001 | 5,339(52.64%) | 4,804(47.36%) |
| Theme 9: pregnancy guidelines | 3542.1 | <.001 | 6,165(82.55%) | 1,303(17.45%) |
| Theme 10: parenting science popularization | 1304.5 | <.001 | 6,971(70.76%) | 2,880(29.24%) |
| Theme 11: growth record | 6228.2 | <.001 | 13,761(92.97%) | 1,040(7.03%) |
| Theme 12: maternal-infant community | 7819.3 | <.001 | 17,199(82.09%) | 3,752(17.91%) |

# Supplementary Table 5 The attribute discrimination index of different factors

| **Factor** | **PSi** | **PCi** | **PCi** | **NCi** | **CDi** | **NTi** | **Attribute** |
| --- | --- | --- | --- | --- | --- | --- | --- |
| Theme 1: system login | 1 | 1 | 0 | 1 | 1 | 0 | Basic factors |
| Theme 2: privacy disclosure | 1 | 1 | 0 | 1 | 1 | 0 | Basic factors |
| Theme 3: storage optimization | 1 | 1 | 1 | 0 | 1 | 1 | Attractive factors |
| Theme 4: page design | 1 | 1 | 1 | 0 | 1 | 1 | Attractive factors |
| Theme 5: function provision | 1 | 1 | 1 | 0 | 1 | 1 | Attractive factors |
| Theme 6: platform feedback | 1 | 1 | 1 | 0 | 1 | 1 | Attractive factors |
| Theme 7: physician’s inquiry | 1 | 1 | 1 | 0 | 1 | 1 | Attractive factors |
| Theme 8: menstrual management | 1 | 1 | 1 | 0 | 1 | 1 | Attractive factors |
| Theme 9: pregnancy guidelines | 1 | 1 | 1 | 0 | 1 | 1 | Attractive factors |
| Theme 10: parenting science popularization | 1 | 1 | 1 | 0 | 1 | 1 | Attractive factors |
| Theme 11: growth record | 1 | 1 | 1 | 0 | 1 | 1 | Attractive factors |
| Theme 12: maternal-infant community | 1 | 1 | 1 | 0 | 1 | 1 | Attractive factors |

# Supplementary Figure 1 Graphical representation of LDA-based model


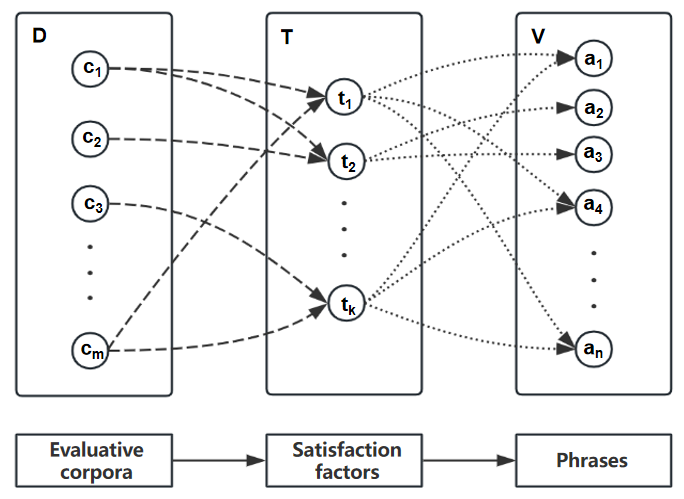


Note: Consider a corpus D, whose elements are individual user reviews retrieved from the initial database search. For this corpus, the set V of unique words defines the vocabulary vector of the corpus. For each word in the vocabulary, its frequency of occurrence in each document is known. Latent Dirichlet Allocation (LDA) is a text mining method based on probabilistic generative models, which uses a three-layer Bayesian framework to analyze latent semantic features in unstructured data. It assumes that documents {c_1_, c_2_,... c_m_} ∈ D, which are composed of a set of words {a_1_, a_2_,... a_n_} ∈ V from the word library. This algorithm optimizes the probability distribution parameters of topic words and document topics through reverse inference, and uses Gibbs sampling or variational inference to achieve semantic deconstruction of large-scale corpora. {t_1_, t_2_,... t_m_} ∈ T represents the topic generated by clustering.

# Supplementary Figure 2 The attribute division basis of the KANO model


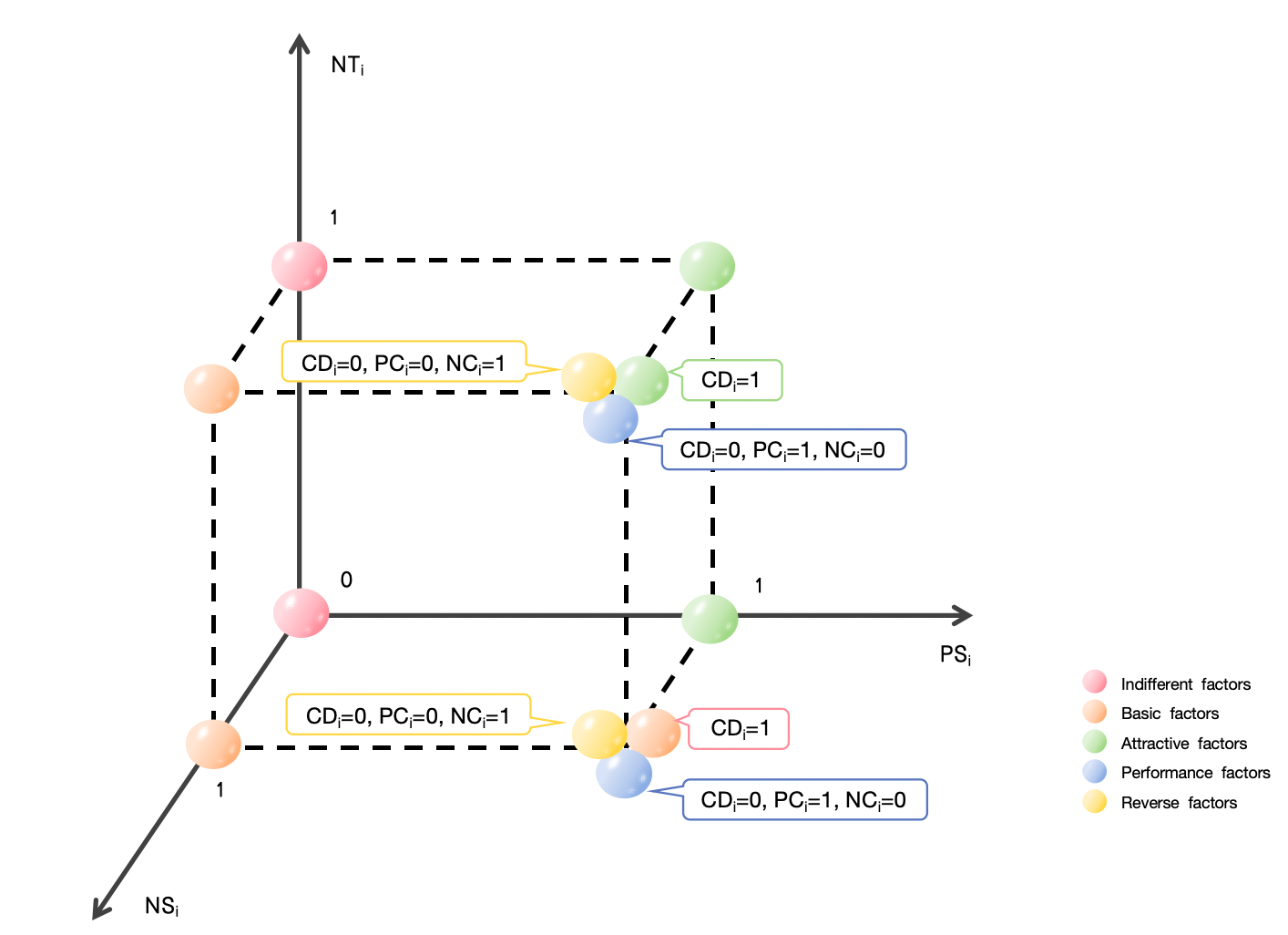


Note: The study introduced six discriminant indicators to classify the factors. PS_i_ indicates whether the influence of factor X_i_ on PD is significant, while NS_i_ indicates whether the influence of factor X_i_ on ND is significant. PC_i_ indicates whether the influence coefficient of X_i_ on PD is positive, while NC_i_ indicates whether the influence coefficient of X_i_ on ND is positive. CD_i_ indicates whether the difference in the influence coefficient of X_i_ on PD and ND is significant, while NT_i_ indicates whether the number of user reviews of X_i_ in PD is greater than that in ND. The specific basis for judgment is as follows:

(1) Indifferent factors. Indifferent factors have no influence on the user satisfaction and dissatisfaction. Whether such factors are provided or not will not change the satisfaction and dissatisfaction of users. When the influencing factors of user satisfaction have no significant effect on the PD and ND, this factor is classified as an indifferent factor. That is, $\mathrm{PS}_{i}$=0, $\mathrm{NS}_{i}$ =0, as shown in the pink ball.

(2) Basic factors. Basic factors are only related to the user's dissatisfaction. The absence of such factors will significantly increase the user's dissatisfaction level, but providing such factors will not improve the user's satisfaction level. In both of the following two cases, the influencing factors can be classified as basic factors, as shown by the orange balls. 1) This factor significantly affects the ND of users but has no significant effect on the PD. That is,$\mathrm{PS}_{i}$=0, $\mathrm{NS}_{i}$=1; 2) This factor has a significant impact on both the PD and ND simultaneously. There is a significant difference in the coefficients of the PD and ND, and the number of negative reviews is greater than that of positive reviews. That is,$\mathrm{PS}_{i}$=1, $\mathrm{NS}_{i}$=1, $\mathrm{CD}_{i}$=1, $\mathrm{NT}_{i}$=0.

(3) Attractive factors. Attractive factors are only related to the user's satisfaction. Such factors can increase the user's satisfaction level, but have little impact on dissatisfaction. In both of the following two cases, the influencing factors of user satisfaction can be classified as charm factors, as shown by the green ball. 1) This factor significantly affects the PD of users but has no significant effect on the ND. That is, $\mathrm{PS}_{i}$=1,$\mathrm{NS}_{i}$ =0; 2) This factor has a significant impact on both the PD and ND of users simultaneously. There is a significant difference in the coefficients of PD and ND, and the number of positive reviews is greater than that of negative reviews. That is, $\mathrm{PS}_{i}$=1,$\mathrm{NS}_{i}$=1, $\mathrm{CD}_{i}$=1, $\mathrm{NT}_{i}$=1.

(4) Performance factors. Performance factors simultaneously affect users' satisfaction and dissatisfaction, and the influences are positively symmetrical. Providing such factors can significantly improve the satisfaction level of users, while the absence of such factors significantly increases the dissatisfaction level of users. When a influencing factor of user satisfaction has a significant impact on the PD and ND, and there is no significant difference in the coefficients of PD and ND, the coefficient of PD is positive and the coefficient of ND is negative, this factor is classified as a performance factor. That is, $\mathrm{PS}_{i}$=1, $\mathrm{NS}_{i}$=1, $\mathrm{CD}_{i}$=0, $\mathrm{PC}_{i}$=1, $\mathrm{NC}_{i}$=0, as shown in the blue ball.

(5) Reverse factors. Reverse factors simultaneously affect the satisfaction and dissatisfaction of users, and the influence on both is inversely symmetrical. Providing such factors will significantly increase the dissatisfaction level of users, while the absence of such factors will significantly increase the satisfaction level of users instead. When a influencing factors has a significant impact on the PD and ND, and there is no significant difference in the coefficients of PD and ND, the coefficient of PD is negative while the coefficient of ND is positive, this factor is classified as a reverse factor. That is, $\mathrm{PS}_{i}$=1, $\mathrm{NS}_{i}$=1, $\mathrm{CD}_{i}$=0, $\mathrm{PC}_{i}$=0, $\mathrm{NC}_{i}$=1, as shown by the yellow ball.

# Supplementary Figure 3 The number of downloads and reviews of pregnancy management apps


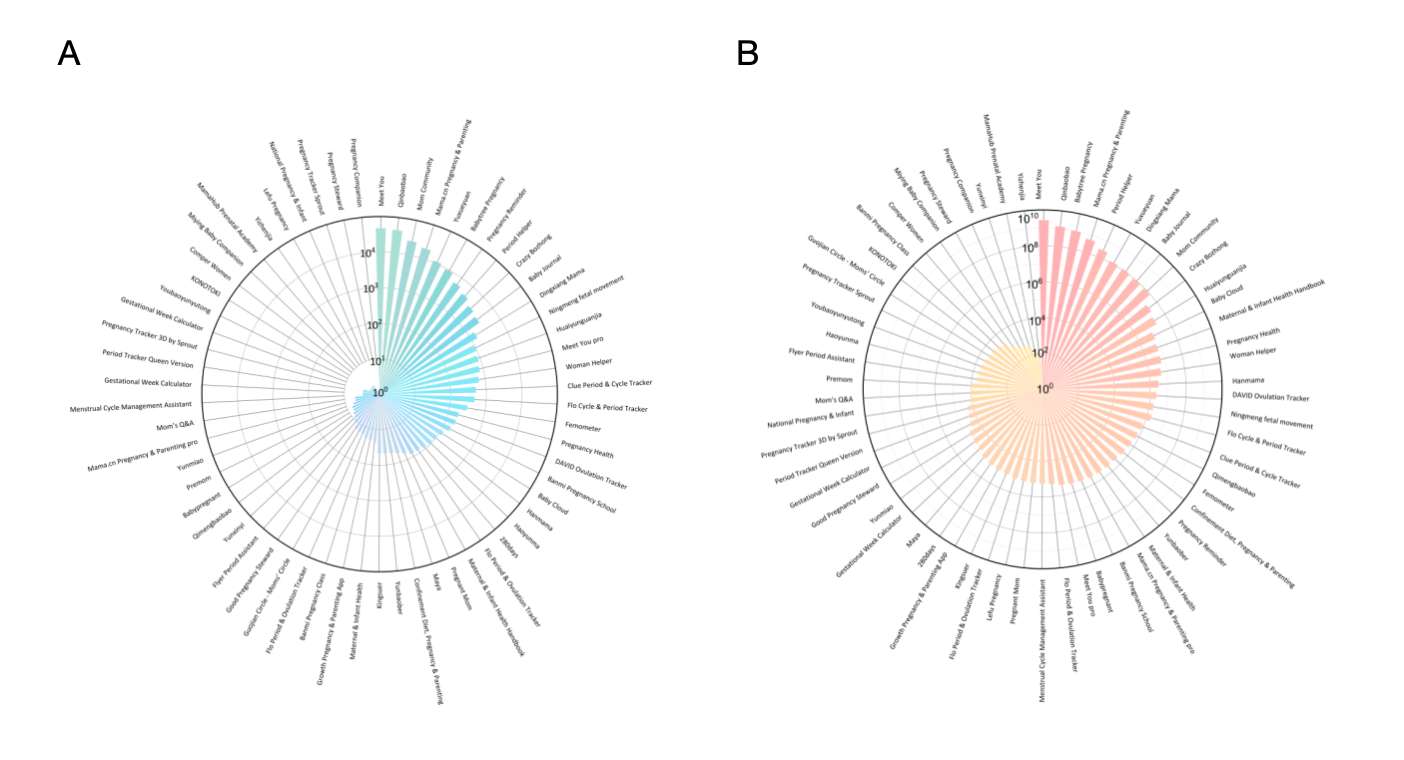


Note: (A) shows the number of user downloads of the pregnancy management apps. (B) shows the number of user reviews.
